# Methods for Measuring T-Cell Memory to Vaccination: From Mouse to Man

**DOI:** 10.3390/vaccines6030043

**Published:** 2018-07-21

**Authors:** Amy Flaxman, Katie J. Ewer

**Affiliations:** The Jenner Institute, University of Oxford, Old Road Campus Research Building, Oxford OX3 7DQ, UK; katie.ewer@ndm.ox.ac.uk

**Keywords:** T-cell, memory, vaccine, pre-clinical, clinical trial

## Abstract

The development of effective vaccines continues to be a key goal for public health bodies, governments, funding bodies and pharmaceutical companies. With new vaccines such as Shingrix targeting Shingles and Bexsero for Meningitis B, licensed in recent years, today’s population can be protected from more infectious diseases than ever before. Despite this, we are yet to license vaccines for some of the deadliest endemic diseases affecting children, such as malaria. In addition, the threat of epidemics caused by emerging pathogens is very real as exemplified by the 2014–2016 Ebola outbreak. Most licensed vaccines provide efficacy through humoral immunity and correlates of protection often quantify neutralising antibody titre. The role of T-cells in vaccine efficacy is less well understood and more complex to quantify. Defining T-cell responses which afford protection also remains a challenge, although more sophisticated assays for assessing cell-mediated immunity with the potential for higher throughput and scalability are now available and warrant review. Here we discuss the benefits of multiparameter cytokine analysis and omics approaches compared with flow cytometric and ELISpot assays. We also review technical challenges unique to clinical trial studies, including assay validation across laboratories and availability of sample type. Measuring T-cell immunogenicity alongside humoral responses provides information on the breadth of immune responses induced by vaccination. Accurately enumerating and phenotyping T-cell immunogenicity to vaccination is key for the determination of immune correlates of protection. However, identifying such T-cell parameters remains challenging without a clear understanding of the immunological mechanisms by which a T-cell-mediated response induces protection.

## 1. Introduction

The implementation of routine vaccination programmes across the globe in the last 70 years has been a major factor in mortality reduction associated with infectious disease. Whilst smallpox is the only infectious disease to have been eradicated through vaccination, the current anti-polio campaign will hopefully do likewise for poliomyelitis in the coming years. Vaccination programmes against other infectious diseases such as measles, influenza, meningitis and yellow fever have afforded markedly reduced mortality rates from these diseases compared with the pre-vaccination era, although these diseases are still prevalent within populations. More recently, the use of vaccines in outbreak situations such as the 2014–2016 Ebolavirus disease (EVD) outbreak in West Africa [1] and from May 2018 the use of vaccination to help prevent the spread of another EVD outbreak in DRC [2] have highlighted the importance of vaccines for the prevention of future epidemics.

Most licensed vaccines mediate protection by eliciting functional antibody responses against the pathogen in question. As a result, assays such as ELISA (or neutralisation assays) which measure immunoglobulin titre are used for licensure and are standardised [3]. However, producing efficacious vaccines against more complex pathogens, especially intracellular bacteria such as *Salmonella enterica*, viruses such as HIV and parasites such as malaria, has proven challenging. For such pathogens, functional antibody is insufficient to provide protection and, therefore, a T-cell vaccine, which induces a memory response able to kill infected cells, is an attractive alternative or complementary approach [4]. Generating antigen-specific CD8^+^ T-cells, which kill cells infected by the pathogen against which vaccination is targeted, is a major goal. However, the “helper” role of CD4^+^ T-cells in antibody production and cytokine production is also important, and must also be quantified in T-cell vaccine development [3,4]. Recently, cytotoxic CD4^+^ T-cells, which kill target cells in an MHC-restricted fashion, have been identified in both mouse and humans with chronic viral infections [5]; therefore, this T-cell subset is also important.

Immunology as a science in its own right was established around 100 years after the advent of vaccinology [6]. In recent years, basic research in deciphering the cellular and humoral immune responses has advanced our knowledge of immunology. Despite this, cellular immune responses required for T-cell vaccine efficacy are difficult to define as T-cell phenotype and patterns of cytokine secretion which lead to efficacy are not well understood. In addition, mechanisms by which long term cellular immunity are maintained are not fully understood; perhaps involving the recruitment of naïve cells rather than the division of existing memory T-cells [7,8,9].

Therefore, selecting T-cell readouts to measure in pre-clinical vaccine studies and clinical trials to assess vaccine immunogenicity and efficacy requires careful consideration. Identification of T-cell-mediated surrogates of protection, where a certain quantifiable parameter correlates with protective efficacy through an unknown protective response, would aid progress of T-cell vaccines, such as those for HIV [10] and TB [11], toward licensure. However, identifying such parameters remains challenging without a clear understanding of the immunological mechanisms by which a T-cell-mediated response induces protection [12]. T-cell phenotype, breadth of response, cytokine secretion, cytotoxic killing and proliferation ability are typical parameters measured in vaccine studies. One way to identify and better understand the cell-mediated immune response to vaccination and pinpoint responses that correlate with efficacy is to use novel methods to assess immunogenicity in pre-clinical studies and to transfer these assays into clinical trials. Such methods can utilise a system’s biology approach, using ‘omics’ rather than focusing on single cytokine responses [11].

Here we review techniques to assess memory T-cell responses to vaccination. We discuss the technical challenges in the assessment of vaccine-induced T-cell immunogenicity from preliminary pre-clinical studies to late stage clinical trials: from mouse to man.

## 2. Memory T-Cells

Much of our knowledge regarding memory T-cell subsets has been derived from mouse studies over the past two decades [13]. Upon antigen exposure, naïve T-cells differentiate into central memory (T_CM_) within lymphoid tissues and effector memory (T_EM_) within peripheral tissues. Both T_CM_ and T_EM_ subsets circulate in the blood [14]. Tissue resident memory T-cells (T_RM_) have been suggested to differentiate from T_EM_ and are found in non-lymphoid tissues such as skin, intestine and liver. These memory T-cell subsets have been extensively reviewed elsewhere [15,16,17] and it is beyond the scope of this review to discuss them in further detail here. 

## 3. T-Cell-Inducing Vaccines

Ideally, a T-cell vaccine would induce long-lived memory T-cells in sufficient numbers and of a correct phenotype to enable clearance of the pathogen before it causes severe disease. Defining what memory T-cell response is required for inducing protection using vaccine X is challenging. Whilst T_EM_ and T_CM_ subsets can be assessed after vaccination, the threshold which may be required for protection is unknown. Evidence for T_RM-_mediated protection to viral infections is mounting [13], so this subset may be a key target to induce through vaccination. Examples of memory T-cell populations supporting protection in natural immunity and in vaccine-induced immunity are numerous. We will discuss a select few here. 

T-cell-mediated protection has been demonstrated in one licensed vaccine, BCG [18]. However, this protection is relatively short lived and so new vaccines against TB are needed. A novel vaccine, L91 was evaluated in mice and when combined with BCG in a prime-boost schedule, L91 elicited greater expansion of T_EM_ and T_CM_ populations as well as boosting efficacy compared to BCG alone [19]. Increased natural CD8^+^ T-cell responses to conserved internal viral antigens were associated with less severe illness during the 2009 H1N1 influenza pandemic [20]. Animal models have shown the importance of T-cells in controlling influenza infection and they can provide heterosubtypic protection [21]. Measles live attenuated vaccine, when administered to 6-month old infants, induces IFN-γ CD4^+^ in the absence of antibody responses which may help to protect against measles-associated mortality and morbidity [22]. As yet, no vaccine in human clinical trials has demonstrated protective efficacy through T-cell immunity alone.

### 3.1. Immunogenicity Requirements for Vaccine Licensure

In the era of reverse vaccinology, we are yet to license a vaccine which elicits protection through T-cell mediated immunity alone. However, vaccines licensed in recent years and those close to licensure measure cellular immunology as part of their development in clinical trials, as recommended by both WHO and EMA guidelines [23,24]. Therefore, what aspects of T-cell memory have been used in progress towards licensure of these vaccines?

Shingrix is a novel VZV glycoprotein E subunit vaccine approved for use in adults over 50 in US and Canada in October 2017 [25]. Most recently, and leading to licensure, Shingrix was shown to protect adults over the age of 70 against herpes zoster and postherpetic neuralgia. In this study, Shingrix induced CD4^+^ T-cell responses expressing at least two activation markers at 36-months which were seven-fold higher than at baseline. [26]. Earlier trials assessing safety and immunogenicity of Shingrix showed that it elicited higher levels of CD4^+^ cells expressing at least two activation markers up to 12 months post vaccination, compared to non-vaccinated controls [27,28].

RTS,S is the leading vaccine candidate for malaria. Despite being trialled extensively over the last 10 years in Africa, it has not yet been licensed, mainly due to the fact that it provides only partial protection [29]. Despite this, it is now being piloted as a routine vaccination in three countries in West Africa, to assess feasibility and safety, with a view to licensure in the next few years [30]. Cell-mediated immunogenicity induced by RTS,S has been assessed after vaccination, where it has been shown to induce antigen-specific CD4^+^ cells expressing at least two activation markers. This same cell subset was higher in vaccine recipients who were protected from infection after challenge [31]. Despite the increased reporting of T-cell responses in vaccines recently licensed or close to licensure, T-cell parameters have not yet been used in the licensure process itself.

### 3.2. Factors to Consider in the Design of Vaccine Studies to Assess T-Cell Memory

Various factors must be considered when planning T-cell immunogenicity assessments post-vaccination. All of the factors listed here can impact establishment and/or measurement of T-cell memory to vaccination and may be dependent on the disease against which we are trying to protect. These factors have been reviewed comprehensively elsewhere as described in Table 1.

## 4. Methods to Assess T-Cell Memory in Vaccine Studies

Extensive reviews on how to perform T-cell assays to assess vaccine immunogenicity have been published previously [39,40] and, therefore, we will not discuss these methods in detail. Instead we will describe briefly the advantages and disadvantages of well-established methods used in vaccine studies and explore more thoroughly newer technologies which are currently being used in, and which could be applied to, vaccine studies.

### 4.1. Advantages and Disadvantages of Established Methods to Assess T-Cell Immunogenicity

The ELISpot assay is a gold standard method for quantifying antigen-specific cellular responses after vaccination in clinical trials [41]. The most widely used ELISpot in vaccine studies is the IFN-γ assay. Advantages of this assay include: It is high throughput, robust and economical, it can be easily standardised and validated, it can be adapted to use in pre-clinical i.e. mouse or non-human primate (NHP) studies and in human trials, both cells and supernatant can be recovered for further analysis, data is obtained from single cells, fine epitope mapping can be performed, and data analysis is straightforward [39,42]. Disadvantages include the lack of information about cell phenotype and the fact that it is normally a single parameter (IFN-γ) readout. Two adaptations to the traditional IFN-γ ELISpot can be used to circumvent these disadvantages; the cultured ELISpot and fluorospot respectively [43]. The cultured ELISpot involves 10 days culture of lymphocytes in the presence of peptide and Il-2, compared with the standard ex vivo 18 h culture and, therefore, the cultured assay is thought to assess mainly T_CM_ [44]. Dual and triple colour fluorospot assays allow analysis of multiple cytokines, typically IFN-γ and IL-2, and commercial kits are available. However, they are more costly than standard ELISpot and require an automated reader with the relevant filters [45]. One application of the ELISpot into a diagnostic test is the T-SPOT, which is used in TB diagnosis and vaccine development to screen for TB [46].

Intracellular cytokine staining (ICS) and analysis by flow cytometry provides an advantage over ELISpot as they allow both multiparameter cytokine analysis and phenotyping from potentially millions of single cells [47]. Typically CD4^+^ and CD8^+^ T-cell populations are assessed along with cytokine expression allowing quantification of subsets, such as % of cytokine positive CD4^+^ cells, which is not achievable by ELISpot. Disadvantages of ICS and flow cytometry compared with ELISpot are that it is much more labour intensive, costs more per sample, requires fixation of cells such that they cannot be used for further assays, requires expensive specialist equipment to run samples, complex panel optimisation, more detailed data analysis using specific software, and is more difficult to validate [48]. Over 10 years ago, Seder and colleagues reviewed Th1 memory and implications for vaccine development, recommending the analysis of IFN-γ, TNF-α, and IL-2 by multiparameter flow cytometry to assess both effector and memory T-cells [49]. Additional cell surface markers can be utilised to assess memory phenotypes, for example CD45RO or CD45RA to phenotype naïve and memory T-cells in human PBMCs, and CCR7 or CD62L to differentiate between T_CM_ and T_EM_ [50]. CD69, an activation marker, can be used alongsideCD103 to identify T_RM_ in both mice and man [51].

**Figure 1:**
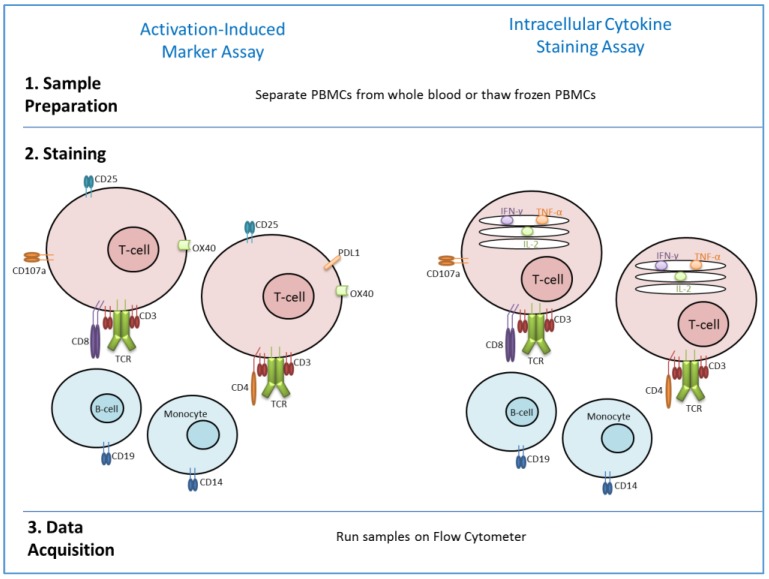
Comparison of methodology for Activation-induced marker assay (as described in [54]) and ICS.

Another flow cytometry-based assay, which has recently been described, is the activation-induced marker (AIM) assay. This assay can be performed in a similar way to ICS (see Figure 1). However, rather than staining for intracellular cytokines, staining is performed for T-cell receptor-induced cell-surface markers including PDL1, CD25 and OX40. Different combinations of these markers were assessed for their ability to identify AIM^+^ T-cell populations [52]. Therefore, detection of antigen-specific T-cells is not dependent upon cytokine production, which may be very low in some T-cell subsets. The assay was initially developed to detect T-follicular helper cells, which are difficult to detect by ICS [53], but can also be used for CD4^+^ T-cell assessment after exposure to antigen by vaccination or infection [52,53]. The use of an AIM assay in a vaccine clinical trial setting showed comparable sensitivity and specificity to ELISpot and ICS [54]. This study showed that AIM assays can be used to detect both CD4^+^ and CD8^+^ T-cells. Whilst CD8^+^ AIM responses correlated with CD8^+^ ELISpot and ICS responses, CD4^+^ AIM responses did not. This is likely due to the fact that AIM assays detect more CD4^+^ subpopulations, including Tregs and NKTs, compared with ICS which primarily detects CD4^+^ T_EM_. This study indicates that AIM assays can be used in vaccine studies to detect novel memory T-cell responses which are hard to enumerate by ICS alone.

### 4.2. Multiplex Cytokine Analysis

Whilst both ELIspot and ICS have a clear role in quantifying T-cell responses, there are a range of newer tools which can be applied to assess T-cell responses to vaccination, both pre-clinically and in clinical trials. A number of these tools assess multiple parameters, rather than one, two or three cytokines. The datasets generated by such analyses require more detailed analysis compared to ELISpot or triple cytokine analysis by ICS, however, the data generated by such analyses can provide much greater information about vaccine responses and helps to generate a bigger picture.

Here we discuss three platforms which measure soluble analytes, essentially providing a multiplex ELISA. Luminex, LegendPlex and Meso-Scale Discovery (MSD) systems can all be used to quantify cytokine levels in cell culture supernatant, tissue homogenate, plasma or serum. A summary of the technical aspects of each of these platforms is provided in Table 2. In terms of T-cell responses after immunisation, multiple cytokine analysis of supernatant from PBMCs stimulated with vaccine antigen peptides, such as that from ELISpot assay, would provide a valuable dataset. We also discuss two platforms based on flow cytometry, which analyse markers in cellular samples, CHIP Cytometry and CyTOF. A summary of the technical aspects of both of these platforms is provided in Table 3.

#### 4.2.1. Luminex

Luminex assays allow multiple cytokine analysis using different fluorescent (red and infrared) beads. Each bead subset is conjugated to capture an antibody specific for a cytokine, chemokine or disease biomarker. Samples such as serum or cell supernatant, are added, and the relevant analyte binds to the relevant antibody coated bead. Biotinylated detection antibody, also specific for the relevant analyte, is applied. The reporter, streptavidin conjugated PE, is then added. Samples are run on a dedicated machine which allows detection of the analyte through the bead fluorescence and quantity of the analyte through PE detection [55]. A number of companies offer kits and machines to allow assessment of cytokines via Luminex, including MerckMillipore, Bio-Rad and ThermoFisher Scientific. Depending on the instrument and kit used, a maximum of 50–80 cytokines can be investigated. Luminex technology has been used in pre-clinical and clinical studies to aid exploration of cytokine profiles induced by vaccination. For example, King and colleagues describe different cytokine patterns induced by co-administration through different immunisation routes of an HIV vaccine in mice. These results indicate that different routes of administration could be used to elicit the desired cellular response from the vaccine [56]. During the development of a vaccine for grass-pollen allergy, cytokine profiles in PBMCs from allergy sufferers were compared after stimulation with the vaccine proteins compared to grass pollen extract. The vaccine induced reduced release of pro-inflammatory cytokines compared to pollen, showing promise for this vaccine in allergy sufferers [57]. Cytokine networks were investigated after vaccination with increasing doses of VSV-ZEBOV in a Phase I trial, and increasing cytokine activity was seen with increasing dose, which will help inform dose selection for future studies [58].

#### 4.2.2. LegendPlex^TM^

The principle behind the LegendPlex^TM^ assay, made by BioLegend, is similar to that of Luminex in that it uses beads with differing levels of APC fluorescence conjugated to an analyte-specific antibody. It also uses biotinylated capture antibody and strepdavidin-PE reagent for detection of a sample. LegendPlex^TM^ also uses two different sized beads, so different analytes are distinguished based on colour and size [59]. Unlike Luminex, the assay does not require a dedicated machine; it can be run on a flow cytometer, although it does require the purchase of specialist analysis software. However, fewer analytes can be multiplexed compared to Luminex platform. This platform provides an attractive alternative to Luminex for a laboratory which already has a dedicated flow cytometer and does not have funds to invest in a new piece of hardware. For example, Copland et al. used LegendPlex^TM^ to assess T-cell cytokine levels in stimulated splenocytes from mice vaccinated with a fusion protein TB candidate. Cytokine levels were higher in mice vaccinated with the new candidate compared with BCG, showing increased immunogenicity of the new vaccine [60].

#### 4.2.3. Meso Scale Discovery

Meso Scale Discovery (MSD) assays work using electrochemiluminescence. Capture antibody is bound to carbon electrodes, sample is applied, and detection antibody conjugated to electrochemiluminescent labels. Samples are run on a dedicated plate reader; electricity is applied to the plate results in light emission from the conjugate, which is measured for quantification. Multiplexing is achieved by having up to 10 spots within the wells of the 96-well plate, each of which has a different detection antibody conjugated to it [61]. A greater number of analytes can be assessed by using multiple plates and plates are customisable. In our experience, MSD assays are much quicker and simpler to run compared with Luminex, especially in terms of the time spent reading plates on the machine; an MSD plate takes only a couple of minutes rather than up to an hour for Luminex. Sample volumes required are similar and cost for a similar number of cytokines is similar. One disadvantage of MSD is that 10 cytokines can be done per plate, so multiplexing of more cytokines requires more plates. Data analysis for MSD is slightly more time consuming compared with Luminex, for which much of the data analysis is done in real time on the machine. One advantage of MSD is that the software is free; so plates can be read and then analysed off site at a later date. Both MSD and Luminex technologies have been validated and compared for human cytokine profiling [62]. MSD technology has been used to assess cytokine responses to influenza vaccination with different adjuvants in a mouse study [63].

#### 4.2.4. CHIP Cytometry

Chipcytometry allows storage of cellular samples in a small chip which can be analysed by a microscopic flow cytometric method at a later date. Cycles of staining, imaging and bleaching allow multiple rounds of analysis, leading to multiplexing [64]. Advantages of this method over flow cytometry include the fact that samples can be stored in their chips for up to 2 years either before analysis or after initial analysis for further analysis at a later date without the loss of biomarkers [65]. This means that samples from a clinical trial could all be biobanked in chips, and all analysed for multiple cytokine and cell immunophenotyping markers, rather than processing each sample on a day-by-day basis, as is the case for many laboratories, with flow cytometry. This technology could have potential in assessing T-cell phenotypes in clinical trials to assess vaccine immunogenicity. It has been used recently in a comparison against flow cytometry methods in assessing cerebrospinal fluid samples from a cohort of patients [66].

#### 4.2.5. CyTOF

Time of flight (TOF) mass cytometry allows the evaluation of more than 40 markers per cell. Instead of labelling with fluorescently conjugated antibodies, cells are labelled with different metal isotope-tagged antibodies. When running samples on the dedicated mass cytometer, each cell is vaporized and metal ions are resolved to produce a mass spectrum. Data from each metal ion relates to the cytokine or cellular parameter in the same way that a detected signal within an emission spectrum does in flow cytometry [67]. One disadvantage of this technology is that it does not have the capacity for forward or side scatter and so cell size and density cannot be assessed. However, there is little compensation required and autofluorescence does not occur. Single cell CyTOF has been used to analyse T-cell functionality in a subset of volunteers in a trial assessing T-cell memory to Hepatitis C virus vaccination. These authors showed a progressive increase in CD8^+^ polyfunctionality post-vaccination. They also validated this CyTOF approach, showing that it correlated to flow cytometric methods [68].

### 4.3. Omics Approaches

Omics approaches have the potential to aid the discovery of novel correlates of protection in combination with traditional analyses such as cytokine levels [69]. Omics technologies add to the “systems vaccinology” approach to enable further understanding of the immune responses to vaccination [70].

DNA and protein microarrays have been used previously for the evaluation of vaccine-specific induced responses [71]. However, these assays require prior knowledge of the protein or DNA targets of interest and technical expertise for assay set up, when compared to RNA-seq or proteomics. For a bacterial pathogen such as TB which expresses thousands of proteins, a protein microarray is a valuable tool [72]. It can be used as a correlate of protection to investigate how responses differ after BCG vaccination [73].

In terms of immunogenicity, RNA-seq analysis post vaccination will add valuable information regarding up and down regulation of parameters other than cytokines and cell surface markers, such as transcription factors, as there is no selection bias—all transcripts within a cell are analysed [74]. However, one challenge with RNA-seq approaches is the skill set and huge time involved in analysing these datasets, compared with cytokine analyses described above. The cost and application of RNA-seq analysis to clinical trial settings is a huge commitment; however, these analyses will become standard in the coming years. Using a single cell RNA-seq approach, Afik and colleagues describe subpopulations of yellow fever virus specific CD8^+^ T-cells exhibiting ‘naïve-like’ and T_EM_ profiles in a volunteer vaccinated against yellow fever [75]. This kind of study allows in-depth analysis of T-cell populations that are not possible though cytokine analyses alone, as other cellular pathways can also be explored. Transcriptional signatures and epigenetics of T-cell memory populations have been described [76]; there are some examples of transcriptomic signatures in vaccine studies which predict vaccine efficacy [77]. Immunopeptidomics, which involves analysing the peptides presented by MHC molecules using mass spectrometry, could help identify T-cell antigens for inclusion in new vaccines and T-cell subsets generated by particular vaccines [78].

## 5. Quantifying T-Cell Memory at Different Stages of Vaccine Development

Many of the methods to assess T-cell memory discussed above can be used in both pre-clinical studies and in the clinic. Pre-clinical vaccine development typically involves immunogenicity testing in one or two animal species, often mouse and NHPs. Challenge studies, where a model is available, give valuable information regarding efficacy. Promising candidates can then move forward into clinical trials. Ideally, approaches for assessing T-cell immunogenicity will be applicable across all the stages of vaccine development, e.g., ex vivo assays such as ELISpot which can be performed on PBMCs after blood sampling. However, there are both technical and logistical aspects to consider.

### 5.1. Pre-Clinical Methods to Better Inform Clinical Trials

Given that so many vaccines are efficacious at the pre-clinical stage, but show lower than expected efficacy in clinical trials, are there new methods which could be utilised at the pre-clinical stage and in early clinical trials to inform about better potential efficacy at the later clinical stage? Limitations at the pre-clinical stage include differences in the markers used in flow cytometry to assess memory T-cell subsets. CD44 can be used in mice to differentiate naïve from T_EM_ and T_CM_, however, CD45RO is used in humans. CD62L can be used in both species to differentiate between T_EM_ and T_CM_ [50,79], and therefore trying to optimise flow cytometry panels which can be used from pre-clinical to clinical stages of vaccine development would be useful.

It is imperative to consider that mice do not replicate the human response to vaccination, and so carefully choosing the mouse model to use in pre-clinical studies is paramount. Many mouse strains are inbred, e.g., BALB/c and C57Bl/6, which are both commonly used to assess vaccine efficacy. Due to their genetic identity, small numbers of these animals can be used in immunogenicity experiments, as the variation throughout the population is low. However, outbred mice, such as CD1 mice, more accurately represent the human population, since they are genetically more heterogeneous. Ideally, vaccine immunogenicity and efficacy should be tested in outbred mice [80], although larger group numbers are required. Immunodominant epitopes to particular vaccine antigens exist in inbred strains of mice. For example, the Pb9 epitope found in *Plasmodium berghei* circumsporozoite protein (CSP) is MHC restricted by H-2K^d^ in Balb/c. Vaccination with CSP in Balb/c mice induces protective CD8^+^ responses [81]. Whilst Pb9 is a useful model epitope in the malaria mouse model, it cannot be used to predict antibody-mediated protection against malaria in humans for CSP vaccines [29].

One example of promising pre-clinical T-cell data in a vaccine study which has aided the progression to clinical trials is the heterosubtypic influenza vaccine. The goal of this vaccine is to induce long lived immunity against multiple subtypes of influenza virus. Viral vectored vaccines expressing internal viral antigens, NP and M1, have been developed and elicit potent T-cell responses to the internal antigens and afford partial protection to influenza infection in preclinical studies [82,83]. MVA-NP+M1 has been used in clinical trials to assess dose escalation [84], immunogenicity in different age groups [85], efficacy [86], and its ability to boost seasonal influenza vaccine immunogenicity through a co-administration study [87]. The current study, INVICTUS, will assess the efficacy of the MVA-NP+M1 vaccine co-administered with the seasonal influenza vaccine. Control subjects receive only seasonal influenza vaccine. Two-thousand participants will be recruited locally over two consecutive influenza seasons. T-cell immunogenicity in a subset of 100 volunteers will be assessed by IFN-γ ELISpot and ICS [88].

### 5.2. Limitations of Clinical Studies

#### 5.2.1. Availability of Sample Types

A benefit of pre-clinical studies is that different sites can be sampled, for example, lymph node and spleen can be assessed for T_CM_ and T_EM_ and non-lymphoid tissues can be assessed for T_RM_. In clinical studies, blood is usually drawn and PBMC assessed for memory populations. In terms of vaccine efficacy, the T-cells poised for pathogen clearance are likely to be found outside the periphery, at sites of disease. For pre-clinical studies assessing liver-stage malaria candidates, assessment of the T-cell compartment in the liver is assessed [89]. Translating this into clinical trials will be possible through liver fine needle aspiration, a method which has been used to assess immune phenotype in the liver compared to the periphery [90]. Sampling of other non-peripheral sites in humans is challenging but is more frequently being achieved. Assessment of multiple sites at the pre-clinical stage allows for the comparison between responses seen at different sites. For example, ELISpot and ICS in mouse studies are often performed using splenocytes. Performing such experiments in PBMCs as well, more challenging as cell yield is lower, would allow differences between the periphery and secondary lymphoid organs to be identified.

#### 5.2.2. Quantity of Sample Available

In adult studies, the volume of blood taken for immunogenicity assessment (typically 40–50 mL) is normally more than sufficient to carry out immunogenicity analyses. However, in older adults PBMC yield per ml of blood is often lower compared to younger adults. We found this to be true in our current ongoing trial to assess the efficacy of heterosubtypic influenza vaccine in over 65s [88] compared to other ongoing studies in 18–45 age groups. In such cases, assay limits must be carefully set, for example, the number of CD3^+^ events in flow cytometry must be above the lower quality control limit. At the other end of the age spectrum, blood sampling in paediatric studies often yields just a few millilitres of blood, although numbers of lymphocytes per ml are higher than in adults. Therefore, assays to be performed on these samples must be carefully designed. Bliss and colleagues used small volumes of blood (4 mL) to successfully perform both ELISpot and ICS analysis from PBMCs in children and infants post malaria vaccination [91]. Whole blood ICS assays can also be performed on small volumes of blood, as shown in a BCG vaccination study [92], and can be applied to vaccination studies in neonates and infants [93].

### 5.3. Technical Considerations in Clinical Trials

Pre-clinical and early stage clinical studies may be performed within one laboratory, by one or two operators and often with coordinated time points. Later stage clinical trials are often carried out over many months or years, across multiple sites and with multiple operators. To ensure quality and consistency across a clinical trial a number of factors must be considered before implementation of the trial can begin.

#### 5.3.1. Fresh versus Frozen Samples

In an ideal situation, all samples should be processed fresh. However, due to logistics, PBMC samples are often frozen for analysis at a later date. Whilst this may have some benefit, in that all samples could be processed at the same time, there are also drawbacks. For example, comparison of T-cell responses by ICS to the same vaccine regimen in the same age group across two different trials, performed in different geographical areas, in which one trial used frozen and one fresh PBMCs for the assay, must be done with an element of caution. This is because there is evidence from a malaria vaccine study that antigen-specific IFNγ producing CD4^+^ T-cells were reduced three to five-fold after freeze-thaw compared with fresh. However, CD8^+^ populations remained mostly unaffected [94]. This effect may not be seen for viral antigens, as fresh and frozen PBMCs were used in ELIspot assays in an HIV vaccination study [95]. However, use and comparison of fresh and frozen samples must be considered in vaccine study design.

#### 5.3.2. Performing Assays across Multiple Sites

During later stage clinical trials, studies are likely to be performed in multiple sites or in field sites which do not always have extensive equipment capabilities. In some cases it may be necessary to perform initial sample processing on site or on multiple sites, before freezing samples and shipping them to a centralised location. The benefit here is that all assays can be performed in a centralised laboratory; however, responses from frozen cells may be reduced, compared to using fresh cells, as described above. On the other hand, there are benefits of on-site analysis for the infrastructure and scientific community around the study site. For example, in trials for Ebola, Malaria and TB carried out in at-risk populations, ELISpots are performed fresh at many field sites. If an ELISPot plate reader is available on site, data acquisition and analysis can also be carried out on site. If not, ELISpot plates can be shipped elsewhere for analysis.

For experiments assessing multiple cytokine analysis, where the sample is often frozen prior to analysis regardless of location, analysis in a centralised location may be preferable over analysis in different locations. This is because a study by Crabb Breen and colleagues showed significant differences between laboratories in cytokine analysis from Luminex and MSD platforms [96]. One example of assay standardisation in clinical trials across sites is provided by Kimani et al, describing a multi-centre clinical trial of a candidate malaria vaccine. Trials were performed in Kenya, the Gambia and Oxford with all cellular assays performed ex vivo on site; batch tested reagents were distributed across all sites and data analysis was checked centrally [97]. Extensive scientific capacity building in Africa has meant that most clinical trial field sites could readily obtain the capability to perform ICS and ELISpot assays in situ, if not already available.

#### 5.3.3. Reagents and Equipment

In order to ensure consistency and continuity throughout clinical trials, batch testing of reagents should be performed. Reagents such as peptides for stimulation in ICS and ELISpot, fetal calf serum for preparation of media, ELISpot plates and antibodies for ICS and ELISpot could be considered. Each new batch should be tested alongside a previously tested batch using cells from the same volunteer. The coefficient of variance of the results between the two batches should be within the pre-specified range for the trial for the new batch to be acceptable for use in the trial. This should minimise any differences in T-cell immunogenicity results throughout the duration of the trial.

Appropriate calibration of equipment must also be performed to ensure results are analysed consistently over the course of a clinical trial. For example, an ELISpot plate reader could be calibrated monthly to ensure that machine performance is consistent. The stage and camera should be calibrated each time a batch of plates is read to ensure the area of the wells being read is consistent. The settings used for detecting spots should be consistent. Using a template with pre-defined settings for spot size and intensity will help ensure that data acquisition is performed consistently across a clinical trial.

### 5.4. Assay Validation

All of the technical considerations discussed above must be taken into account when performing assay validation for a clinical trial. One of the key tasks when moving from pre-clinical to clinical trials is whether the assay of choice is validated and whether assay validation needs to be performed. Assay validation is defined as “objective evidence that a method fulfills the requirements for its intended use” and guidance for parameters on which to assess the assay in order to validate are available [98]. The primary endpoint assay in a clinical trial must be validated for GCP compliance. For example, if the primary endpoint assay to measure vaccine immunogenicity is an ELISpot assay, then that assay should be validated.

After validation, an assay can be carried out across multiple sites with greater precision, thus addressing some of the issues raised above regarding sample processing across different laboratories. Quality control of a dataset is imperative before publication of results of a clinical trial. This may involve checking a percentage of the raw data for a study. A number of collaborative organisations, such as FLUCOP [99] and TRANSVAC [100], exist with the aim of harmonising assays across sites and trials. For example, duplicate assays might be performed for one or two samples for submission to a harmonisation body so that they can assess assay quality in different laboratories over time.

### 5.5. Efficacy Studies

One very important question is how does vaccine-induced T-cell immunogenicity relate to efficacy? In pre-clinical studies this can be assessed with adoptive transfer and challenge studies. Challenge studies can also be performed in clinical trials for diseases which have treatments available such as malaria and bacterial infections such as *S. typhi*. In pre-clinical vaccine studies for malaria, IFNγ producing CD8^+^ T-cells were induced and led to protection [101]. A controlled human malaria infection trial carried out within our institute found that protection correlated with antigen-specific IFNγ producing CD8^+^ T-cells after vaccination with viral vectors expressing pre-erythrocytic antigens [102].

### 5.6. Longevity Studies

Phase IV trials aim to assess safety and have a role in pharmacovigilance, once a vaccine is licensed. Other studies carried out post-licensure can assess T-cell immunogenicity in smaller populations. In a recent study, participants received yellow fever vaccine after which deuterium labelling was used to monitor virus-specific CD8^+^ cells longitudinally. This paper utilises some of the methods described above which could be applicable in vaccine development. Transcriptional profiling and epigenetic analysis of naïve CD8^+^ cells and vaccine-specific T-cell subsets longitudinally showed that long-lived vaccine-specific T-cells do not express effector molecules, but their epigenetic signature resembles that of effector populations [103]. Therefore, this suggests that vaccine developers could use similar methods to those described above to evaluate vaccine-specific T-cells post-licensure.

## 6. Conclusions

Whilst the conventional methods for evaluating T-cell responses to vaccination are effective and reliable, they are relatively one-dimensional. Multiparameter analysis is possible and vaccine immunogenicity studies are reporting transcriptional profiling and multiple cytokine analyses with increasing frequency. Progression of vaccine candidates from the laboratory into the clinic and ultimately to the public will be aided by these approaches. To do this, technical aspects such as sample availability and assay validation must be considered. Having the tools in place to identify and evaluate protective memory T-cell responses can accelerate a vaccine from the laboratory to the target population.

## Figures and Tables

**Table 1:** Factors to consider when planning vaccine studies.

Factor	Example	References
Vaccine Platform	Protein, VLP, DNA vaccine, viral vector, use of adjuvant	[32,33,34,35]
Vaccine Regimen	Prime only, Homologous or Heterologous Prime-Boost	[36]
Route of administration	Intramuscular, subcutaneous, intranasal, sublingual, aerosol	[37,38]
Timing of sampling for immunogenicity assessment	Hours, days or weeks post immunisation	[36]

**Table 2:** Comparison of platforms which allow multiplex analysis of soluble samples.

Technical Aspect	Luminex	LegendPlex^TM^	Meso Scale Discovery
Principle of assay	Fluorescent beads	Fluorescent beads	Electrochemiluniscence
Bead based?	Yes	Yes	No
Plate based?	Yes	No	Yes
Species available	Human, mouse, rat, NHP, canine and others	Human, mouse, rat, NHP	Human, mouse, rat, NHP
Maximum Number of Analytes	50–80	13	10 per plate
Minimum Sample volume required	12.5 µL or 25 µL, depending on kit manufacturer	Supernatant—25 µL Serum/plasma—12.5 µL	12.5 µL
Number of tests/samples	39 per plate (performed in duplicate)	100 tests per kit	40 per plate (performed in duplicate)
Estimated sample preparation time including incubations	4–5 h	4–5 h	4.5–5 h
Estimated cost per multiplex plate/kit	£1000–5000	~£1000	£1000–1500
Data Acquisition time	~1 h per plate	As for flow cytometry: 2–3 min per sample plus set up time	<5 min per plate
Dedicated data Acquisition instrument?	Yes, e.g., Magpix, Luminex 200, FLEXMAP 3D	No, Flow cytometer that can detect PE and APC	Yes, SECTOR range of instruments or Quickplex
Data analysis software	xPONENT, usually purchased with instrument	LEGENDplex™ data analysis software—Free Download	MSD Discovery workbench—Free Download

**Table 3:** Comparison of platforms which allow multiplex analysis of cellular samples.

Technical Aspect	CHIP Cytometry	CyTOF
Principle of assay	Flow Cytometry	Mass spectrometry
Species available	Human	Human. Mouse
Number of Analytes	90+	40+
Assay volume/cell number	100 µL per chip	Human PBMC assay: 3 × 10^6^
Dedicated data Acquisition instrument?	Yes, ZellScanner ONE™ (manual) or CYTOBOT (automated)	Yes, Helios or CyTOF^®^ 2 Instrument
Data analysis software	ZellExplorer	Cytobank

## References

[1] Henao-Restrepo A.M., Longini I.M., Egger M., Dean N.E., Edmunds W.J., Camacho A., Carroll M.W., Doumbia M., Draguez B., Duraffour S. (2015). Efficacy and effectiveness of an rvsv-vectored vaccine expressing ebola surface glycoprotein: Interim results from the guinea ring vaccination cluster-randomised trial. Lancet.

[2] Who Supports Ebola Vaccination of High Risk Populations in the Democratic Republic of the Congo. http://www.who.int/news-room/detail/21-05-2018-who-supports-ebola-vaccination-of-high-risk-populations-in-the-democratic-republic-of-the-congo.

[3] Plotkin S.A. (2010). Correlates of protection induced by vaccination. Clin. Vaccine Immunol..

[4] Robinson H.L., Amara R.R. (2005). T cell vaccines for microbial infections. Nat. Med..

[5] Takeuchi A., Saito T. (2017). CD4 CTL, a cytotoxic subset of CD4^+^ T cells, their differentiation and function. Front. Immunol..

[6] Ulvestad E. (2007). Defending Life: The Nature of Host-Parasite Relations.

[7] Borghans J., Ribeiro R.M. (2017). The maths of memory. eLife.

[8] Macallan D.C., Borghans J.A., Asquith B. (2017). Human T cell memory: A dynamic view. Vaccines.

[9] Omilusik K.D., Goldrath A.W. (2017). The origins of memory T cells. Nature.

[10] Koup R.A., Graham B.S., Douek D.C. (2011). The quest for a T cell-based immune correlate of protection against HIV: A story of trials and errors. Nat. Rev. Immunol..

[11] Bhatt K., Verma S., Ellner J.J., Salgame P. (2015). Quest for correlates of protection against tuberculosis. Clin. Vaccine Immunol..

[12] Plotkin S.A. (2013). Complex correlates of protection after vaccination. Clin. Infect. Dis..

[13] Rosato P.C., Beura L.K., Masopust D. (2017). Tissue resident memory T cells and viral immunity. Curr. Opin. Virol..

[14] Sallusto F., Lenig D., Forster R., Lipp M., Lanzavecchia A. (1999). Two subsets of memory T lymphocytes with distinct homing potentials and effector functions. Nature.

[15] Farber D.L., Yudanin N.A., Restifo N.P. (2014). Human memory T cells: Generation, compartmentalization and homeostasis. Nat. Rev. Immunol..

[16] Woodland D.L., Kohlmeier J.E. (2009). Migration, maintenance and recall of memory T cells in peripheral tissues. Nat. Rev. Immunol..

[17] Seder R.A., Ahmed R. (2003). Similarities and differences in CD4^+^ and CD8^+^ effector and memory T cell generation. Nat. Immunol..

[18] Hanekom W.A. (2005). The immune response to BCG vaccination of newborns. Ann. N. Y. Acad. Sci..

[19] Rai P.K., Chodisetti S.B., Zeng W., Nadeem S., Maurya S.K., Pahari S., Janmeja A.K., Jackson D.C., Agrewala J.N. (2017). A lipidated peptide of mycobacterium tuberculosis resuscitates the protective efficacy of BCG vaccine by evoking memory T cell immunity. J. Transl. Med..

[20] Sridhar S., Begom S., Bermingham A., Hoschler K., Adamson W., Carman W., Bean T., Barclay W., Deeks J.J., Lalvani A. (2013). Cellular immune correlates of protection against symptomatic pandemic influenza. Nat. Med..

[21] Clemens E.B., van de Sandt C., Wong S.S., Wakim L.M., Valkenburg S.A. (2018). Harnessing the power of T cells: The promising hope for a universal influenza vaccine. Vaccines.

[22] Gans H.A., Yasukawa L.L., Alderson A., Rinki M., DeHovitz R., Beeler J., Audet S., Maldonado Y., Arvin A.M. (2004). Humoral and cell-mediated immune responses to an early 2-dose measles vaccination regimen in the united states. J. Infect. Dis..

[23] Guideline on Clinical Evaluation of Vaccines. http://www.ema.europa.eu/docs/en_GB/document_library/Scientific_guideline/2018/04/WC500248095.pdf.

[24] Guidelines on clinical evaluation of vaccines: Regulatory expectations. http://www.who.int/biologicals/Clinical_guidelines_revised_IK_29_Oct_2015.pdf.

[25] Ogunjimi B., Beutels P. (2018). Uk experience of herpes zoster vaccination can inform varicella zoster virus policies. Lancet Public Health.

[26] Cunningham A.L., Lal H., Kovac M., Chlibek R., Hwang S.J., Diez-Domingo J., Godeaux O., Levin M.J., McElhaney J.E., Puig-Barbera J. (2016). Efficacy of the herpes zoster subunit vaccine in adults 70 years of age or older. N. Engl. J. Med..

[27] Leroux-Roels I., Leroux-Roels G., Clement F., Vandepapelière P., Vassilev V., Ledent E., Heineman T.C. (2012). A phase 1/2 clinical trial evaluating safety and immunogenicity of a varicella zoster glycoprotein e subunit vaccine candidate in young and older adults. J. Infect. Dis..

[28] Chlibek R., Smetana J., Pauksens K., Rombo L., Van den Hoek J.A., Richardus J.H., Plassmann G., Schwarz T.F., Ledent E., Heineman T.C. (2014). Safety and immunogenicity of three different formulations of an adjuvanted varicella-zoster virus subunit candidate vaccine in older adults: A phase II, randomized, controlled study. Vaccine.

[29] Rts S.C. (2015). Efficacy and safety of rts,s/as01 malaria vaccine with or without a booster dose in infants and children in Africa: Final results of a phase 3, individually randomised, controlled trial. Lancet.

[30] RTS,S. http://www.malariavaccine.org/malaria-and-vaccines/first-generation-vaccine/rtss.

[31] Kester K.E., Cummings J.F., Ofori-Anyinam O., Ockenhouse C.F., Krzych U., Moris P., Schwenk R., Nielsen R.A., Debebe Z., Pinelis E. (2009). Randomized, double-blind, phase 2a trial of falciparum malaria vaccines RTS,S/AS01B and RTS,S/AS02A in malaria-naive adults: Safety, efficacy, and immunologic associates of protection. J. Infect. Dis..

[32] Humphreys I.R., Sebastian S. (2017). Novel viral vectors in infectious diseases. Immunology.

[33] Mohan T., Verma P., Rao D.N. (2013). Novel adjuvants & delivery vehicles for vaccines development: A road ahead. Indian J. Med. Res..

[34] Charlton Hume H.K., Lua L.H.L. (2017). Platform technologies for modern vaccine manufacturing. Vaccine.

[35] Ewer K.J., Lambe T., Rollier C.S., Spencer A.J., Hill A.V., Dorrell L. (2016). Viral vectors as vaccine platforms: From immunogenicity to impact. Curr. Opin. Immunol..

[36] Nolz J.C., Harty J.T. (2011). Strategies and implications for prime-boost vaccination to generate memory CD8 T cells. Adv. Exp. Med. Biol..

[37] Schulze K., Ebensen T., Riese P., Prochnow B., Lehr C.M., Guzman C.A. (2016). New horizons in the development of novel needle-free immunization strategies to increase vaccination efficacy. Curr. Top. Microbiol. Immunol..

[38] Scherliess R. (2011). Delivery of antigens used for vaccination: Recent advances and challenges. Ther. Deliv..

[39] Saade F., Gorski S.A., Petrovsky N. (2012). Pushing the frontiers of T-cell vaccines: Accurate measurement of human T-cell responses. Expert Rev. Vaccines.

[40] Coughlan L., Lambe T. (2015). Measuring cellular immunity to influenza: Methods of detection, applications and challenges. Vaccines.

[41] Ranieri E., Popescu I., Gigante M. (2014). CTL elispot assay. Methods Mol. Biol..

[42] Lehmann P.V., Zhang W., Kalyuzhny A.E. (2012). Unique strengths of elispot for T cell diagnostics. Handbook of Elispot: Methods and Protocols.

[43] Calarota S.A., Baldanti F. (2013). Enumeration and characterization of human memory T cells by enzyme-linked immunospot assays. Clin. Dev. Immunol..

[44] Reyes-Sandoval A., Pearson F.E., Todryk S., Ewer K. (2009). Potency assays for novel t-cell-inducing vaccines against malaria. Curr. Opin. Mol. Ther..

[45] Ahlborg N., Axelsson B., Kalyuzhny A.E. (2012). Dual-and triple-color fluorospot. Handbook of Elispot: Methods and Erotocols.

[46] T-spot. http://www.tspot.com/about-the-test/.

[47] Lovelace P., Maecker H.T., Hawley T.S., Hawley R.G. (2011). Multiparameter intracellular cytokine staining. Flow Cytometry Protocols.

[48] Freer G., Rindi L. (2013). Intracellular cytokine detection by fluorescence-activated flow cytometry: Basic principles and recent advances. Methods.

[49] Foulds K.E., Chang-you W., Seder R.A. (2006). Th1 memory: Implications for vaccine development. Immunol. Rev..

[50] Sallusto F., Geginat J., Lanzavecchia A. (2004). Central memory and effector memory t cell subsets: Function, generation, and maintenance. Annu. Rev. Immunol..

[51] Kumar B.V., Ma W., Miron M., Granot T., Guyer R.S., Carpenter D.J., Senda T., Sun X., Ho S.H., Lerner H. (2017). Human tissue-resident memory T cells are defined by core transcriptional and functional signatures in lymphoid and mucosal sites. Cell Rep..

[52] Reiss S., Baxter A.E., Cirelli K.M., Dan J.M., Morou A., Daigneault A., Brassard N., Silvestri G., Routy J.P., Havenar-Daughton C. (2017). Comparative analysis of activation induced marker (aim) assays for sensitive identification of antigen-specific CD4^+^ T cells. PloS ONE.

[53] Dan J.M., Lindestam Arlehamn C.S., Weiskopf D., da Silva Antunes R., Havenar-Daughton C., Reiss S.M., Brigger M., Bothwell M., Sette A., Crotty S. (2016). A cytokine-independent approach to identify antigen-specific human germinal center t follicular helper cells and rare antigen-specific CD4^+^ T cells in blood. J. Immunol..

[54] Bowyer G., Rampling T., Powlson J., Morter R., Wright D., Hill A.V.S., Ewer K.J. Activation-induced markers detect vaccine-specific CD4^+^ T cell responses not measured by assays conventionally used in clinical trials. Vaccines.

[55] Multiplex Immunoasssays. http://www.bio-rad.com/en-uk/applications-technologies/multiplex-immunoassays?ID=LUSM0E8UU.

[56] King D.F., McKay P.F., Mann J.F., Jones C.B., Shattock R.J. (2015). Plasmid DNA vaccine co-immunisation modulates cellular and humoral immune responses induced by intranasal inoculation in mice. PloS ONE.

[57] Focke-Tejkl M., Weber M., Niespodziana K., Neubauer A., Huber H., Henning R., Stegfellner G., Maderegger B., Hauer M., Stolz F. (2015). Development and characterization of a recombinant, hypoallergenic, peptide-based vaccine for grass pollen allergy. J. Allergy Clin. Immunol..

[58] Dahlke C., Kasonta R., Lunemann S., Krahling V., Zinser M.E., Biedenkopf N., Fehling S.K., Ly M.L., Rechtien A., Stubbe H.C. (2017). Dose-dependent T-cell dynamics and cytokine cascade following rvsv-zebov immunization. EBioMedicine.

[59] Legendplex. https://www.biolegend.com/legendplex.

[60] Copland A., Diogo G.R., Hart P., Harris S., Tran A.C., Paul M.J., Singh M., Cutting S.M., Reljic R. (2018). Mucosal delivery of fusion proteins with bacillus subtilis spores enhances protection against tuberculosis by bacillus calmette-guérin. Front. Immunol..

[61] Our Technology. https://www.mesoscale.com/en/technical_resources/our_technology.

[62] Chowdhury F., Williams A., Johnson P. (2009). Validation and comparison of two multiplex technologies, luminex^®^ and mesoscale discovery, for human cytokine profiling. J. Immunol. Methods.

[63] Magnusson S.E., Reimer J.M., Karlsson K.H., Lilja L., Bengtsson K.L., Stertman L. (2013). Immune enhancing properties of the novel matrix-m™ adjuvant leads to potentiated immune responses to an influenza vaccine in mice. Vaccine.

[64] Hennig C., Adams N., Hansen G. (2009). A versatile platform for comprehensive chip-based explorative cytometry. Cytometry A.

[65] Technology. http://www.zellkraftwerk.com/technology/.

[66] Hummert M.W., Alvermann S., Gingele S., Gross C.C., Wiendl H., Mirenska A., Hennig C., Stangel M. (2018). Immunophenotyping of cerebrospinal fluid cells by chipcytometry. J. Neuroinflammation.

[67] Helios, a Cytof System. https://www.fluidigm.com/products/helios#overview.

[68] Swadling L., Capone S., Antrobus R.D., Brown A., Richardson R., Newell E.W., Halliday J., Kelly C., Bowen D., Fergusson J. (2014). A human vaccine strategy based on chimpanzee adenoviral and mva vectors that primes, boosts, and sustains functional hcv-specific t cell memory. Sci. Transl. Med..

[69] Mooney M., McWeeney S., Canderan G., Sékaly R.-P. (2013). A systems framework for vaccine design. Curr. Opin. Immunol..

[70] Hagan T., Nakaya H.I., Subramaniam S., Pulendran B. (2015). Systems vaccinology: Enabling rational vaccine design with systems biological approaches. Vaccine.

[71] Natesan M., Ulrich R.G. (2010). Protein microarrays and biomarkers of infectious disease. Int. J. Mol. Sci..

[72] Deng J., Bi L., Zhou L., Guo S.-J., Fleming J., Jiang H.-W., Zhou Y., Gu J., Zhong Q., Wang Z.-X. (2014). Mycobacterium tuberculosis proteome microarray for global studies of protein function and immunogenicity. Cell Rep..

[73] Marsay L., Matsumiya M., Tanner R., Poyntz H., Griffiths K.L., Stylianou E., Marsh P.D., Williams A., Sharpe S., Fletcher H. (2013). Mycobacterial growth inhibition in murine splenocytes as a surrogate for protection against mycobacterium tuberculosis (m. Tb). Tuberculosis.

[74] Chattopadhyay P.K., Roederer M. (2015). A mine is a terrible thing to waste: High content, single cell technologies for comprehensive immune analysis. Am. J. Transplant..

[75] Afik S., Yates K.B., Bi K., Darko S., Godec J., Gerdemann U., Swadling L., Douek D.C., Klenerman P., Barnes E.J. (2017). Targeted reconstruction of T cell receptor sequence from single cell rna-seq links cdr3 length to t cell differentiation state. Nucleic Acids. Res..

[76] Youngblood B., Hale J.S., Ahmed R. (2013). T-cell memory differentiation: Insights from transcriptional signatures and epigenetics. Immunology.

[77] Wang I.M., Bett A.J., Cristescu R., Loboda A., ter Meulen J. (2012). Transcriptional profiling of vaccine-induced immune responses in humans and non-human primates. Microb. Biotechnol..

[78] Faridi P., Wayne Purcell A., Croft N.P. (2018). In immunopeptidomics we need a sniper instead of a shotgun. Proteomics.

[79] Bachmann M.F., Wolint P., Schwarz K., Jager P., Oxenius A. (2005). Functional properties and lineage relationship of CD8^+^ T cell subsets identified by expression of il-7 receptor alpha and cd62l. J. Immunol..

[80] Davis H.L. (2008). Novel vaccines and adjuvant systems: The utility of animal models for predicting immunogenicity in humans. Human Vaccines.

[81] Rollier C.S., Hill A.V.S., Reyes-Sandoval A. (2016). Influence of adenovirus and mva vaccines on the breadth and hierarchy of t cell responses. Vaccine.

[82] Lambe T., Carey J.B., Li Y., Spencer A.J., van Laarhoven A., Mullarkey C.E., Vrdoljak A., Moore A.C., Gilbert S.C. (2013). Immunity against heterosubtypic influenza virus induced by adenovirus and mva expressing nucleoprotein and matrix protein-1. Sci. Rep..

[83] Mullarkey C.E., Boyd A., van Laarhoven A., Lefevre E.A., Veronica Carr B., Baratelli M., Molesti E., Temperton N.J., Butter C., Charleston B. (2013). Improved adjuvanting of seasonal influenza vaccines: Preclinical studies of mva-np+m1 coadministration with inactivated influenza vaccine. Eur. J. Immunol..

[84] Berthoud T.K., Hamill M., Lillie P.J., Hwenda L., Collins K.A., Ewer K.J., Milicic A., Poyntz H.C., Lambe T., Fletcher H.A. (2011). Potent CD8^+^ T-cell immunogenicity in humans of a novel heterosubtypic influenza a vaccine, mva-np+m1. Clin. Infect. Dis..

[85] Antrobus R.D., Lillie P.J., Berthoud T.K., Spencer A.J., McLaren J.E., Ladell K., Lambe T., Milicic A., Price D.A., Hill A.V. (2012). A t cell-inducing influenza vaccine for the elderly: Safety and immunogenicity of mva-np+m1 in adults aged over 50 years. PLoS ONE.

[86] Lillie P.J., Berthoud T.K., Powell T.J., Lambe T., Mullarkey C., Spencer A.J., Hamill M., Peng Y., Blais M.E., Duncan C.J. (2012). Preliminary assessment of the efficacy of a t-cell-based influenza vaccine, mva-np+m1, in humans. Clin. Infect. Dis..

[87] Antrobus R.D., Berthoud T.K., Mullarkey C.E., Hoschler K., Coughlan L., Zambon M., Hill A.V., Gilbert S.C. (2014). Coadministration of seasonal influenza vaccine and mva-np+m1 simultaneously achieves potent humoral and cell-mediated responses. Mol. Ther..

[88] Improved Novel Vaccine Combination Influenza Study (Invictus). https://clinicaltrials.gov/ct2/show/NCT03300362.

[89] Spencer A.J., Longley R.J., Gola A., Ulaszewska M., Lambe T., Hill A.V. (2017). The threshold of protection from liver-stage malaria relies on a fine balance between the number of infected hepatocytes and effector CD8^+^ T cells present in the liver. J. Immunol..

[90] Pembroke T., Gallimore A., Godkin A. (2015). Tracking the kinetics of intrahepatic immune responses by repeated fine needle aspiration of the liver. J. Immunol. Methods.

[91] Bliss C.M., Bowyer G., Anagnostou N.A., Havelock T., Snudden C.M., Davies H., de Cassan S.C., Grobbelaar A., Lawrie A.M., Venkatraman N. (2018). Assessment of novel vaccination regimens using viral vectored liver stage malaria vaccines encoding me-trap. Sci. Rep..

[92] Hanekom W.A., Hughes J., Mavinkurve M., Mendillo M., Watkins M., Gamieldien H., Gelderbloem S.J., Sidibana M., Mansoor N., Davids V. (2004). Novel application of a whole blood intracellular cytokine detection assay to quantitate specific t-cell frequency in field studies. J. Immunol. Methods.

[93] Nemes E., Hesseling A.C., Tameris M., Mauff K., Downing K., Mulenga H., Rose P., van der Zalm M., Mbaba S., Van As D. (2018). Safety and immunogenicity of newborn mva85a vaccination and selective, delayed bacille calmette-guerin for infants of human immunodeficiency virus-infected mothers: A phase 2 randomized, controlled trial. Clin. Infect. Dis..

[94] Ford T., Wenden C., Mbekeani A., Dally L., Cox J.H., Morin M., Winstone N., Hill A.V.S., Gilmour J., Ewer K.J. (2017). Cryopreservation-related loss of antigen-specific ifngamma producing CD4^+^ T-cells can skew immunogenicity data in vaccine trials: Lessons from a malaria vaccine trial substudy. Vaccine.

[95] Jaoko W., Karita E., Kayitenkore K., Omosa-Manyonyi G., Allen S., Than S., Adams E.M., Graham B.S., Koup R.A., Bailer R.T. (2010). Safety and immunogenicity study of multiclade hiv-1 adenoviral vector vaccine alone or as boost following a multiclade hiv-1 DNA vaccine in Africa. PLoS ONE.

[96] Breen E.C., Reynolds S.M., Cox C., Jacobson L.P., Magpantay L., Mulder C.B., Dibben O., Margolick J.B., Bream J.H., Sambrano E. (2011). Multisite comparison of high-sensitivity multiplex cytokine assays. Clin. Vaccine Immunol..

[97] Kimani D., Jagne Y.J., Cox M., Kimani E., Bliss C.M., Gitau E., Ogwang C., Afolabi M.O., Bowyer G., Collins K.A. (2014). Translating the immunogenicity of prime-boost immunization with chad63 and mva me-trap from malaria naive to malaria-endemic populations. Mol. Ther..

[98] Andreasson U., Perret-Liaudet A., van Waalwijk van Doorn L.J., Blennow K., Chiasserini D., Engelborghs S., Fladby T., Genc S., Kruse N., Kuiperij H.B. (2015). A practical guide to immunoassay method validation. Front. Neurol..

[99] Flucop. http:www.flucop.eu.

[100] Transvac. http:www.transvac.org.

[101] Epstein J.E., Tewari K., Lyke K.E., Sim B.K., Billingsley P.F., Laurens M.B., Gunasekera A., Chakravarty S., James E.R., Sedegah M. (2011). Live attenuated malaria vaccine designed to protect through hepatic CD8^+^ T cell immunity. Science.

[102] Ewer K.J., O’Hara G.A., Duncan C.J., Collins K.A., Sheehy S.H., Reyes-Sandoval A., Goodman A.L., Edwards N.J., Elias S.C., Halstead F.D. (2013). Protective CD8^+^ T-cell immunity to human malaria induced by chimpanzee adenovirus-mva immunisation. Nat. Commun..

[103] Akondy R.S., Fitch M., Edupuganti S., Yang S., Kissick H.T., Li K.W., Youngblood B.A., Abdelsamed H.A., McGuire D.J., Cohen K.W. (2017). Origin and differentiation of human memory CD8 T cells after vaccination. Nature.

